# Systematic review and meta-analysis: relative age in attention-deficit/ hyperactivity disorder and autism spectrum disorder

**DOI:** 10.1007/s00787-024-02459-x

**Published:** 2024-05-20

**Authors:** Eleni Frisira, Josephine Holland, Kapil Sayal

**Affiliations:** Institute of Mental Health, School of Medicine, Mental Health and Clinical Neurosciences, University of Nottingham, Innovation Park, Triumph Road, Nottingham, NG7 2TU UK

**Keywords:** ADHD, Autism, Relative age

## Abstract

Youngest students in their class, with birthdates just before the school entry cut-off date, are overrepresented among children receiving an Attention-Deficit/Hyperactivity Disorder (ADHD) diagnosis or medication for this. This is known as the relative age effect. This systematic review and meta-analysis summarises the evidence on the influence of relative age on ADHD symptoms, diagnosis and medication prescribing. As no review to date has investigated the association with autism spectrum disorder (ASD) diagnosis, this is also examined. Following prospective registration with PROSPERO, we conducted a systematic review according to the PRISMA guidelines. We searched seven databases: Medline, Embase, PsycInfo, Web of Science Core Collection, ERIC, Psychology and Behavioural Sciences Collection, and Cochrane Library. Additional references were identified from manual search of retrieved reviews. We performed a meta-analysis of quantitative data. Thirty-two studies were included, thirty-one investigated ADHD and two ASD. Younger relative age was associated with ADHD diagnosis and medication, with relative risks of 1.38 (1.36–1.52 95% CI) and 1.28 (1.21–1.36 95% CI) respectively. However, risk estimates exhibited high heterogeneity. A relative age effect was observed for teacher ratings of ADHD symptoms but not for parent ratings. With regard to ASD, the youngest children in their school year were more likely to be diagnosed with ASD. This review confirms a relative age effect for ADHD diagnosis and prescribed ADHD medication and suggests that differences in teacher and parent ratings might contribute to this. Further research is needed on the possible association with ASD.

**Supplementary Information:**

The online version contains supplementary material available at 10.1007/s00787-024-02459-x.

## Introduction

Relative age refers to the age difference between children grouped together in the same school year due to the school entry cut-off date. The youngest students in the school year have birthdates just before the cut-off date while their older peers have birthdates up to 12 months earlier [1, 2]. Children of younger relative age are expected by adults to match the educational and behavioural expectations of their relatively older classmates. Although there are interindividual differences in maturation besides relative age, younger students are likely to be less developmentally mature than their older classmates [3]. Previous systematic reviews and meta-analyses summarising data from different countries, have shown that youngest students in their school year are overrepresented among children with an Attention-Deficit/Hyperactivity Disorder (ADHD) diagnosis or receiving ADHD medication [3–6], a phenomenon referred to as the relative age effect (RAE). ADHD is a neurodevelopmental condition, usually diagnosed in childhood, characterised by developmentally inappropriate levels of motor hyperkinesis, impulsivity and difficulties in attention and organisation [7, 8]. Although the prevalence of ADHD varies globally, with administrative clinical diagnosis rates ranging from around 2% to 7% [9], the RAE has been observed in both countries with lower and higher prescribing rates of ADHD medication [3, 4]. Given that most countries have different cut-off dates for school entry with differing policies, international comparisons can be helpful in understanding the underlying reasons for diagnostic and prescribing variations observed with relative age [3, 10, 11].

ADHD assessment and diagnosis is a multi-step process, requiring an evaluation by a clinician, observational reports from school professionals on behavioural or academic problems and input from parents [12]. In addition to studies using administrative record data from population-wide databases, teacher and parent ratings of ADHD symptoms have been increasingly reported in literature [13]. Accounts from teachers and parents on ADHD-related symptoms are highly relevant due to their essential contribution to the diagnostic process, providing insight into the child’s symptoms and functional difficulties in multiple settings [14, 15]. Understanding the extent of the RAE on teacher and parent reporting of children’s symptoms is important in the assessment process for children with possible ADHD [13] and so this review aims to expand the scope to explore the role of different informants in the relative age effect phenomenon, an area not extensively investigated in previous reviews.

The RAE in ADHD has been widely studied; however, the effect on autism spectrum disorder (ASD) diagnosis much less extensively investigated [16]. Children who are relatively younger in the school year may show variation in language skills and be less likely to meet the social demands of their classroom [17], both of which could resemble common features of ASD [18].

The topic of the relative age effect remains a rapidly progressing area of international research, with a continuously expanding body of evidence. This systematic review summarises the evidence on how being relatively younger in the school year could affect three domains: (1) ratings of ADHD symptoms by teachers or parents, (2) receiving a diagnosis of ADHD and (3) receiving ADHD medications; aiming to quantify this effect wherever possible using meta-analysis. We hypothesised that the relative age effect may have become attenuated over recent years as clinicians diagnosing and prescribing medications for ADHD may have been more aware of the relative age phenomenon, therefore taking a child’s relative age into account as part of their assessment. Additionally, this review broadens the scope by including ASD as a comparator neurodevelopmental condition to provide a more comprehensive understanding of the relative age effect across different diagnostic categories. We hypothesised that the effect would be observable within the context of ASD.

## Methods

### Registration

This systematic review was prospectively registered with PROSPERO (ID: CRD42022373175) and was conducted in accordance with the PRISMA reporting guidelines [19]. (see online resource 1).

### Search

Two separate searches for primary studies investigating the effect of relative age within a school year on symptoms, diagnosis or prescription of medication or ADHD or diagnosis of ASD were conducted. They were searched on the 23rd August 2022 in the following databases: MEDLINE® ALL, Embase, PsycInfo (via Ovid), the Cochrane Central Register of Controlled Trials (CENTRAL) and the Cochrane Database of Systematic Reviews (through the Cochrane Library), Web of Science Core Collection, ERIC, and Psychology and Behavioral Sciences Collection (via EBSCOhost). The search strategies were undertaken by an information specialist using free text terms (searching the title and abstract) as well as advanced search syntax (truncation, Boolean logic AND/OR, and proximity searching) to ensure all relevant studies were identified. Relevant controlled vocabulary headings for each database were searched and relevant terms were identified. The search terms included the following themes, with synonyms to describe each: relative age and ADHD (see online resource 2); relative age and autism spectrum disorder (see online resource 3). Additionally, a manual search of the references of systematic reviews and meta-analyses retrieved in the database search was conducted for any possible studies missed by the database search.

### Study selection

Rayyan software was used to assist with study selection. Duplicates were removed and two independent reviewers (EF, JH) screened the titles and abstracts of the search results; 100% consensus was reached between reviewers (initial 2% discrepancy resolved with discussion). Full text assessments were completed independently by EF and JH, who agreed on final paper inclusion (7% discrepancy resolved with discussion to reach 100% consensus).

### Inclusion criteria

There were no date restrictions, and all observational studies were eligible for inclusion if they reported a measure of ADHD symptoms, diagnosis or prescription of medication, or ASD symptoms or diagnosis, for participants up to 18 years of age, in relation to their age within a school year, including month (either recorded grouped months or month) of birth. For studies measuring prescribed medications for ADHD, this was used as a proxy for confirmed clinical diagnosis.

### Exclusion criteria

Publications were excluded if they: (1) were review papers or meta-analyses, (2) were case or conference abstracts with no corresponding full text-paper for retrieval, (3) were not available in the English language, (4) included adult populations only, (5) did not report month (or grouped months) of birth of participants in relation to school entry, (6) commented only on effect of relative age on symptoms or behaviours not directly relevant to ADHD or ASD. Grey literature was not searched.

### Data extraction

For the included studies, the following data were extracted on Microsoft excel: authors, year of publication, country of study, total sample size, years studied, the cut-off date for school entrance in the source population, age range of studied population, data source, reported socio-demographic characteristics, the time period/calendar month used as exposure measure in each study between younger and older children, the number of children with symptoms, the symptom measure used, who rated the symptoms, and/or clinical diagnosis and/or being prescribed medication for ADHD or ASD (absolute number or risk ratios as available). If a study met inclusion criteria but did not provide sufficient data for analysis, the authors were contacted once to provide additional information.

### Risk of bias

The risk of bias was assessed by EF for each study included using the Newcastle–Ottawa Quality Assessment tool [20]. The Newcastle–Ottawa Scale (NOS) measures the selection, comparability and outcome measures of the included studies, and is rated out of 9 in total. Not all domains were applicable to all studies and so some studies were rated out of lower total scores. Studies scoring between 7 and 9 were considered of high methodological quality, between 5 and 7 of moderate and below 5 of low quality. A full breakdown of NOS scores for individual studies is available in online resource 4. The authors have no conflicts of interest.

### Data analysis and synthesis

We conducted a narrative synthesis of the available evidence on the relative age effect on ADHD symptoms, diagnosis or prescribed medication, and ASD, and as far as possible quantitatively describe these relationships using meta-analysis. Meta-analysis was performed using Review Manager version 5.3, with a random effects model due to the expected heterogeneity of data based on previous meta-analyses [3]. Missing data were excluded from the data synthesis and all materials used in the review are available upon request. Outcome measures are presented as relative risk (RR) with 95% confidence intervals.

## Results

**Fig. 1:**
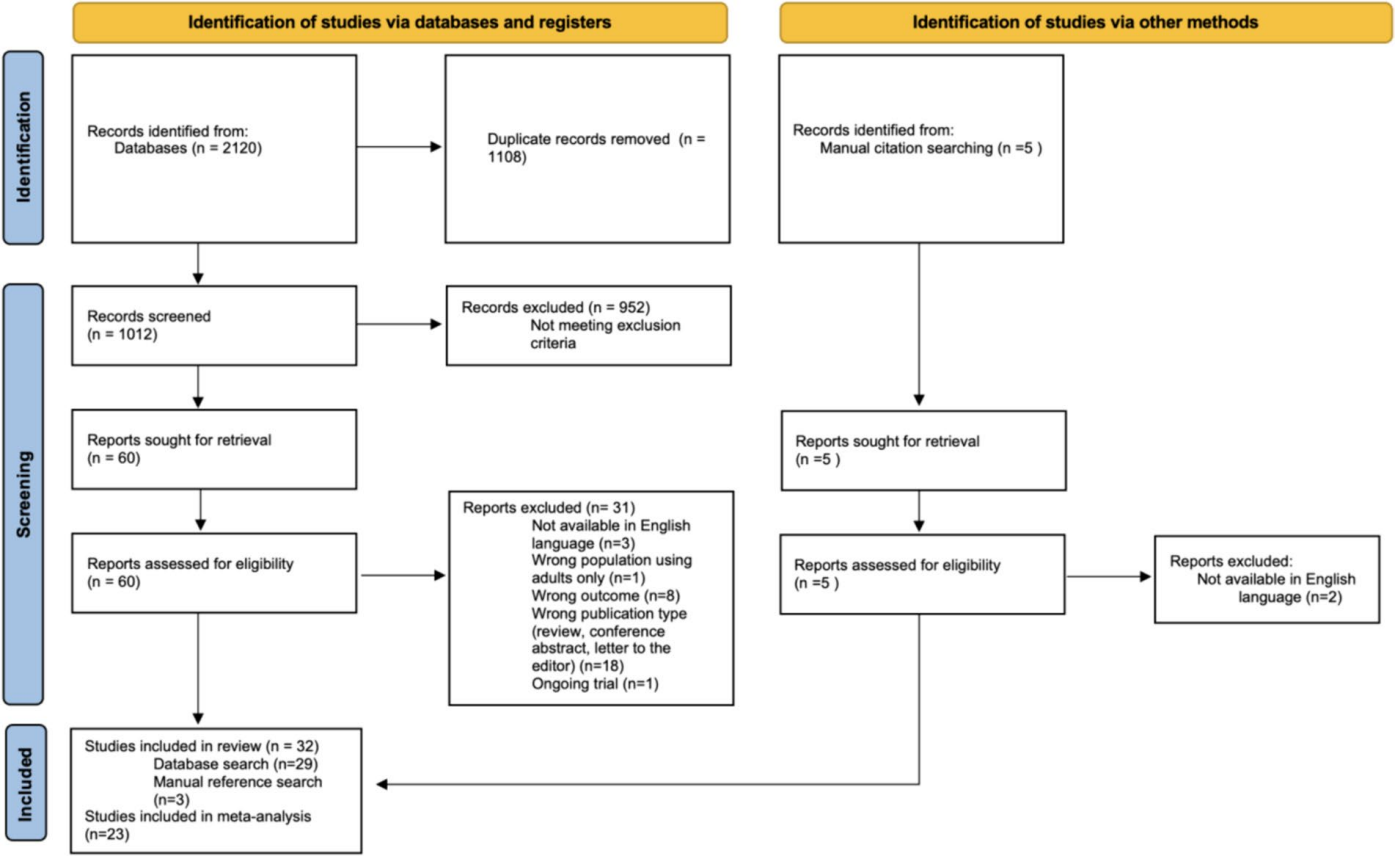
Prisma flow diagram

A total of 2120 papers were retrieved in the database search, leaving 1012 papers for screening after duplicates were removed (Fig. 1). Following initial title and abstract screening, 60 full-text papers were assessed for eligibility, of which 31 were excluded. Five further papers were identified from manual citation search, three of which were included. In total, 32 papers were included in the review. Study characteristics are presented in Table 1. Most included studies examined the role of relative age and ADHD (n = 31) with only two investigating ASD. Due to the low number of studies on ASD, no meta-analysis was possible, although a narrative review is presented.

**Table 1:** Summary of characteristics and main results of included studies investigating symptoms, diagnosis and prescribed medication for ADHD

Study	Years studied	Sample size (n)	Country	Data source	Reported Social, Demographic Characteristics^a^	Ages studied	School starting age/ Cut-off date	ADHD Symptoms/ Diagnosis/Medication	Symptoms measure	Clinical diagnosis definition	Medication definition	Time Comparison	RAE (Relative Age Effect)	Strength of RAE	NOS^b^
Bruno (2022) Nation-wide arm of study	2013–2017	400,098	Australia	National medicine dispensing dataset for a 15% random sample of Australian children receiving medicines	Not reported^c^	4–9	Calendar year of 5th birthday (6th in Tasmania) Cut-off varies by territory In 4/ 6 territories they can delay entry by a year	Medications	n/a	n/a	Initiation of prescription for medications approved in Australia for ADHD (including clonidine as off-license)	4-month blocks	Yes	Specific incidence ratios presented per jurisdiction in original paper, ranging from 1.2 to 2.8 in girls and 0.9–1.5 in boys. (Adjusted^d^)	7/8
Bruno (2022) Delayed entry adjusted arm of study	2009–2012	150,906	Australia	New South Wales (NSW) Australian Early Development Census (AEDC)	Not reported^c^	4–9	Calendar year of 5th birthday, cut-off date July 31st (they can delay entry by a year)	Medications	n/a	n/a	Initiation of prescription for medications approved in Australia for ADHD (including clonidine as off-license)	Monthly	Opposite effect	Incidence rate ratios for girls 0.7 [95%CI 0.24–1.75] and for boys 0.5 [95% CI 0.29–0.78] when compared with the oldest children (who had delayed school entry by 1 year) (Adjusted^d^)	7/8
Whitely (2017)	2013	311,384	Australia	Pharmaceutical Benefits Scheme Register	Not reported	6–15	Cut-off June 30th	Medications	n/a	n/a	At least one prescription for ADHD on Pharmaceu-tical Benefits Scheme register	1 month either side of cut- off date	Yes	“The RR for children born in June were 1.52 [95% CI, 1.30–1.73] for boys and 1.73 [95% CI, 1.42–1.94] for girls compared to born in July.” (Adjusted^d^)	6/8
Brault (2022)	2017–2019	1,294	Canada	17 elementary public schools from the province of Québec	50.2% males^c^	4–12	Calendar year of 5th birthday, cut-off date September 30th	Symptoms, Diagnosis and Medications	Parent and teacher rated unspecified inattention and hyperactivity scale	Parent reported clinical ADHD diagnosis	Parent reported ADHD medication prescription	Monthly	Yes for diagnosis Yes for teacher rated symptoms No for parent rated symptoms No for medication	“OR for ADHD diagnosis for younger students is 1.05 [95% CI 1.01–1.10] p = 0.023” “OR 3.03 [95% CI 1.81–5.07] of being suspected of ADHD by teachers”, p < 0.001but not parents, p = 0.117 “RAE was not statistically significant for taking ADHD medication, p = 0.058.” (Adjusted^d^)	6/9
Furzer (2022)	1994–2003	7,510	Canada	National Longitudinal Survey of Child and Youth	50% males	5–12	Cut-off varies by province	Symptoms	Parent and teacher rated CBS^e^	n/a (diagnosis was considered against school starting age but not relative age)	n/a	1 month either side of the cut-off date	Yes for teachers No for parental hyperactivity scores	“Younger children within school year were rated by teachers to have 0.58 units higher hyperactivity and 0.88 units higher inattention scores, p < 0.01. No statistically significant difference for hyperactivity scores rated by parents. Younger children within school year were rated by parents to have 0.26 higher inattention scores.” (Adjusted^d^)	5/9
Morrow (2012)	1997–2008	937,943	Canada	Health Databases: Pharmanet, Medical Services Plan, Canadian Institute for Health information Discharge Abstracts Database	Not reported	6–12	Calendar year of 6th birthday, cut-off date December 31st	Diagnosis and Medications	n/a	Administrative record, not otherwise specified	1 or more prescription of ADHD medication	1 month either side of cut-off	Yes	“Boys born in December had RR 1.30, [95% CI 1.23–1.37] and girls RR 1.70 [95% CI 1.53–1.88] for ADHD diagnosis compared to those born in January.”“Boys had RR 1.41, [95% CI 1.33–1.50] and girls had RR 1.77, [95% CI 1.57–2.00] for ADHD medication if born December compared to January.” (Adjusted^d^)	7/8
Dalsgaard(2012)	1990–2001	416,744	Denmark	Danish Psychiatric Central Register	51% males	> 7	Calendar year of 7th birthday, cut-off date 1st of January	Diagnosis	n/a	Administrative data of clinician diagnosis	n/a	100 days either side of cut-off	No	No RAE seen	6/7
Dalsgaard(2014)	1990–2001	418,396	Denmark	Danish Civil Registration System	Not reported^c^	> 7	Calendar year of 7th birthday	Medication	n/a	Medication as proxy for diagnosis	Purchase of ADHD medication	Linear regression	No	No RAE seen	7/8
Pottergard(2014)	2000–2012	932,032	Denmark	Danish National Prescription Registry, Danish Student Register, Danish Civil Registration System	Not reported	7–12	Calendar year of 7th birthday	Medications	n/a	Medication as proxy for diagnosis	1 or more prescription	1 month and 2 months either side of cut-off date, 3-month blocks	No	No RAE seen in either time block	7/8
Sayal (2017)	1998–2011	6,136 cases (no controls)	Finland	Finnish Hospital Discharge Register (FHDR)	Not reported	> 7	Calendar year of 7th birthday	Diagnosis	n/a	Administrative record of clinician diagnosis based on ICD-10	n/a	4-month blocks	Yes	“The incidence ratio of ADHD diagnosis for the youngest boys and girls in the school year was 1.26 [95% CI 1.18–1.35] and 1.31 [95% CI1.12–1.54], p = 0·0007.” (Adjusted^d^)	7/8
Vuori (2020)	2005–2017	182,802	Finland	National Prescription Register	Not reported	6–12	Calendar year of 6th birthday, cut-off December 31st	Medications	n/a	n/a	ADHD prescription medication when the prescription indication is ADHD	4-month blocks	Yes	Relatively younger boys had HR 1.3 [95%CI 1.1–1.5] of ADHD medication prescriptions and girls had HR ranging from 1.4–1.7. (Adjusted^d^)	6/8
Diefenbach (2021)	2014–2015	1,633	Germany	79 primary schools in the German Federal State of Rhineland-Palatinate	51.9% males	6–7	Calendar year of 6th birthday, cut-off date September 1st	Diagnosis and Symptoms	Parent and teacher rated hyperactivity/ inattention scales of SDQ^f^	Parent reported clinical ADHD diagnosis	n/a	4 months either side of cut-off date	Yes for parent and teacher rated symptoms No for ADHD diagnosis	“A one-year increase in age at school entry significantly reduced the odds of suspected ADHD by up to 75% (parent reports) or 54% (teacher reports).” (Adjusted^d^)	8/9
Schwandt (2015)	2008–2011	7,200,000	Germany	Social health insurance records (90% of population)	Not reported	4–14	Calendar year of 6th birthday, cut-off date varies by state	Diagnosis and Medications	n/a	Administrative record of clinical diagnosis based on ICD-10	1 or more prescription of ADHD medication	Monthly	Yes	“Children born right before the cut-off have up to one percentage point higher ADHD rates compared to those born right after the cut-off.” “Prescription of ADHD medication increased about 0.8 percentage points” for children before the cut-off compared to the month after. (Adjusted^d^)	8/8
Zoega (2012)	2003–2009	11,785	Iceland	Icelandic Medicines Registry	Not reported	9–12	Calendar year of 6th birthday, cut-off January 1st	Medications	n/a	Medication as proxy for diagnosis	1 or more prescription	4-month blocks	Yes	“Children in the youngest third of class were 50% more likely [95% CI 28%–80%] than those in the oldest third to be prescribed stimulants for ADHD.” (Adjusted^d^)	6/8
Hoshen (2016)	2006–2011	1,013,149	Israel	Clalit Health Services- Health Insurance Data (covering 50% of population)	51% males 56% Jewish, 39% Arab, 5% Ultra-Orthodox Jewish 53% low SES^g^, 14% high SES^g^	6–17	Calendar year of 6th birthday, cut-off December	Medication	n/a	Medication as proxy for diagnosis	Re-imbursement of ADHD medications	4-month blocks	Yes	“The youngest third of children in class was more likely to be prescribed medication than the oldest third RR 1.17, [95% CI1.12–1.23] or the middle third RR 1.06 [95% CI 1.01–1.11].” (Adjusted^d^)	8/9
Bonati (2018)	2011–2017	4,070	Italy	Regional ADHD Registry	86% males	> 6	Calendar year when child turns 6	Diagnosis	n/a	Clinical diagnosis based on 7 step diagnostic evaluation for children with suspected and reported ADHD	n/a	4-month blocks	Yes	“The incidence ratio of ADHD diagnosis for those born September–December (youngest) was 1.54 [95% CI 1.13–2.09] p < 0.0058 compared to those born in January – April.” (Adjusted^d^)	6/8
Karlstad (2017)	2004–2014	509,827	Norway	Norwegian Patient Registry, Norwegian Directorate of Health, and Norwegian Prescription Database	Not reported^c^	6–14	Calendar year of 6th birthday, cut-off date December 31st	Diagnosis and Medications	n/a	Administrative record of ICD clinical diagnosis (hyperkinetic disorders)	1 or more dispensed ADHD medications	3-month blocks	Yes	For diagnosis, HR was 1.5 [95% CI1.4–1.5] for boys in the youngest 3-month group and for girls HR was 1.8 [95% CI 1.6–1.9]. For medication, HR for boys born in the last 3 months of the year was 1.4 [95% CI 1.4–1.5] and for girls 1.8 [95% CI 1.7–2.0]. (Adjusted^d^)	7/8
Fleming (2022)	Scotland (2009– 2013) and Wales (2009– 2016)	1,063,256	Scotland and Wales	Prescribing Information System for Scotland and Welsh Demographic Service (WDS) for Wales	50% males, 23% most deprived SES^i^, 18% least deprived	Not specified	Cut-off In Scotland March 1st and in Wales September 1st In Scotland, they may delay entry by a year	Medications	n/a	n/a	Receipt of one or more medication licensed solely for ADHD	3-month blocks	Yes in WalesYes in Scotland when held-back children were adjusted for	In Wales, children in the youngest quartile had OR 1.32 [95% CI 1.19–1.46] compared to oldest one. In Scotland, the prevalence of ADHD medications increased from the oldest quartile to the second youngest, but then fell in the youngest quartile with no statistically significant difference than oldest one Held-back children were more likely to have treated ADHD (Scotland OR 2.18, 95% CI 2.01–2.36; Wales OR 1.70 [95% CI 1.21–2.31]. (Adjusted^d^)	7/8
Halldner (2014)	2005–2009	10,760	Sweden	Child and Adolescent Twin Study in Sweden (CATSS)	50% males	9	Cut-off January 1st	Symptoms	Parent rated A-TAC^h^ (> 4 cut-off for ADHD symptoms)	n/a	n/a	2 months either side of cut-off date	No	No RAE seen	7/8
Halldner (2014)	2005–2009	41,452 for diagnosis 45,762 for meds	Sweden	Swedish Total Population, Migration and Cause of Death Registers, National Patient Register (NPR) and Prescribed Drug Register (PDR)	50% males	6–17	Cut-off date January 1st	Diagnosis and Medications	n/a	Administrative record of ICD clinical diagnosis (hyperkinetic disorders)	1 or more dispensed prescription of ADHD medications	2 months either side of cut-off date	Yes	“The OR for ADHD were significantly higher (ORs 1.1–1.6)” for children born in November/ December compared to January/ February”ADHD medication prescribing was significantly higher (ORs 1.2–1.8)” for children born in November/ December compared to January/ February. (Adjusted^d^)	7/8
Kuntsi (2022)	2005–2013	297,840	Sweden	National Patient Register (NPR) and Prescribed Drug Register (PDR)	Not reported^c^	15–24	Cut-off date January 1st	Diagnosis	n/a	Administrative record of ICD-9 or- 10 clinical diagnosis or medications as proxy	n/a	2 months either side of cut-off date	Yes	“ADHD prevalence was higher in children with young relative age (2.8%) compared to those with older relative age (1.7%), p < 0.001.” (Adjusted)	5/8
Chen (2016)	1997–2011	378,881	Taiwan	National Health Insurance Research Database (NHIRD)	Not reported	4–17	Cut-off date 31st August	Diagnosis and Medications	n/a	Administrative record of clinician diagnosis (given at least twice by board certified psychiatrists)	1 or more prescription of ADHD medication	1 month either side of cut-off date	Yes	“Children born in August had a higher risk of being diagnosed with ADHD, OR 1.63 [5% CI 1.45–1.84], OR 1.71 [95% CI 1.36–2.15] and receiving ADHD medication, OR 1.76 [95% CI 1.53–2.02], OR 1.65 [95% CI 1.26–2.18] than those born in September.” (Adjusted^d^)	7/8
Chen (2021)	2001–2011	9,548,393	Taiwan	National Health Insurance Research Database (NHIRD)	Not reported^c^	3–17	Cut-off date August 31st	Diagnosis and Medications	n/a	Administrative record of ICD-9 clinical diagnosis	ADHD medications, methylphenidate or atomoxetine as reported on national register	3-month blocks	Yes	Comparing children in the youngest 3-month group to the oldest 3-month group, OR for ADHD was 1.55 [95% CI 1.52–1.57] and for ADHD meds 1.59 [95% CI 1.57–1.62]. (Adjusted^d^)	7/8
Hsu (2021)	1995–2013	1,951,777	Taiwan	National Health Insurance Research Database (NHIRD)	Not reported	Not specified	Cut-off date August 31st	Diagnosis	n/a	DSM-5 diagnosis of ADHD by a board-certified psychiatrist	n/a	3-month blocks	Yes	Children who were relatively young had a RR range of 1.07 to 1.29 of ADHD compared to relatively old children, RR 0.81–0.98. (Adjusted^d^)	7/8
Krabbe (2014)	Not specified	2,218	The Netherlands	GPs that are registered with a province health care support organisation	Not reported	5–12	Calendar year of 5th birthday, cut-off September 1st	Medications	n/a	Medication as proxy for diagnosis	Methylphenidate prescription on GP record	Monthly	Yes	The youngest children in class have a 2.43 greater likelihood of being prescribed methylphenidate compared to the oldest children in class [95% CI 1.09–5.42]. (Unadjusted)	4/9
Wienen (2018)	2009–2015	1,973	The Netherlands	29 primary schools	51% males	6–12	Year they are six years old on the 1st of October	Symptoms	Teacher rated hyperactivity/ inattention scale of SDQ^f^	n/a	n/a	Continuous linear regression model	No	No RAE seen	6/9
Gökçe (2017)	Not specified	3,969	Turkey	Public primary schools in the Kadkoy county of Istanbul	48% males	First and second grade	Between 60–66 months of age, no cut-off date specified	Symptoms	Teacher rated SNAP-IV^i^, CTRS^j^, and PCS^k^	n/a	n/a	Comparison made based on school starting age, not relative age within schoolyear	Yes	“Beginning primary school at younger ages OR 5 0.95 [95% CI 0.92–0.97], p < 0.001 was significantly associated with probable ADHD.” (Adjusted^d^)	5/9
Oner (2019)	Not specified	2,627	Turkey	10 private primary schools in Istanbul	55% males All participants attended private schools	5–9	Between 60–72 months depending on parental preferences	Symptoms	Teacher rated SNAP-IV^i^	n/a	n/a	Younger than the 25th vs older than the 75th percentiles for consecutive grades	Yes	“ADHD-symptoms were 2.5–3.6 times higher in children in youngest quartile.” (Adjusted^d^)	4/6
Broughton (2022)	1991–2013	14,643	UK	Avon Longitudinal Study of Parents and Children	51% males 95% White ethnicity	4–25	Cut-off date September 1st	Symptoms	Parent rated hyperactivity sub-scale of SDQ^f^	n/a	n/a	4 weeks either side of the cut-off date	Yes	“A 1-year decrease in relative age in the school year was associated with a difference of 0.25 [95% CI 0.18- 0.32] in SDQ hyperactivity subscale difficulties.” (Adjusted^d^)	8/9
Root (2019)	2012–2017	1,042,106	UK	Clinical Practice Research Datalink (CPRD)	51% males 70% White, 6% South Asian, 4.3% Black, 2% Other, 2% Mixed, 15% missing, 9% least deprived SES^j^, 11% most deprived SES	4–15	August 31 in England and Wales, February 28 in Scotland, and July 1 in Northern Ireland	Diagnosis	n/a	Administrative record of clinical diagnosis or medication prescription record	n/a	3-month blocks	Yes	Children born in the last quarter of the school year (youngest within their year) were “1.35 [95% CI 1.27–1.45] times more likely to be diagnosed with ADHD”. (Adjusted^d^)	7/8
Elder (2010)	1998–2007	11,784	United States	Data from National Centre for Education Statistics longitudinal survey	56% White, non-Hispanic	Not specified	Calendar year of 6th birthday, cut-off date varies by state	Symptoms, Diagnosis and Medications	Teacher and parent rated ADHD- related symptoms, unspecified scales	Parent reported clinician diagnosis	Parent reported ADHD medication	50 days either side of cut-off date	Yes	“60% increase among those born in the month immediately after cut-off” (Unadjusted)	5/8
Evans (2010)	NHIS 1996–2006, MEPS 1997–2006, Private 2003–2006	NHIS 35,343, MEPS 31,641, Private 18,559	United States	National Health Interview Survey (NHIS) -diagnosis, Medical Expenditure Panel Survey (MEPS) -medications, Private health insurance company -medications	51% males 64.6% White non-Hispanic, 15.4%Black 15.4%, 15.7% Hispanic, 4.4% Other ethnicity	7–17	Calendar year of their 6th birthday (5th for kindergarten), cut-off date varies by state	Diagnosis and Medications	n/a	Parent- reported clinician diagnosis	1 or more prescription of ADHD medication	4 months either side of the cut-off date	Yes	“Children born before the cut-off have a 9.7% diagnosis rate compared with only 7.6% for those born after.” “Stimulant usage has a 0.5 percentage point difference” between children born before and children born after the cut-off date. (Adjusted^d^)	5/8
Schneider (2006)	2002	9,278	United States	Early Childhood Longitudinal Survey	51% males 62.5% White non-Hispanic, 20% Hispanic, 9% Black, 3% Asian	Third grade	Calendar year of 6th birthday, cut-off varies by state but most September 1st	Diagnosis	n/a	Parent- reported clinician diagnosis	n/a	3-month blocks	Yes	“Children born in the summer were more likely to have an ADHD diagnosis OR: 1.69 [95% CI 1.10–2.61]” (Adjusted^d^)	5/8

^a^There was no consistency in the reported sociodemographic characteristics of the total sample of included studies. ^b^NOS Newcastle Ottawa Score. ^c^Authors adjusted their analysis for some sociodemographic characteristics. ^d^Authors adjusted for variables of their choice. List of individual variables adjusted for in each study can be found in individual papers. ^e^CTRS Conner’s Teacher Rating Scale. ^f^SDQ Strengths and Difficulties Questionnaire. ^g^SES Socioeconomic status. ^h^A-TAC Autism – Tics, ADHD and other Comorbidities Inventory. ^i^SNAP-IV Swanson, Nolan, and Pelham –IV Questionnaire. ^j^PCS Perceived Competence Scale. ^k^CBS Children’s Behavioural Scale

## ADHD

### ADHD symptom ratings

Nine studies measured ratings of ADHD symptoms, three of which provided ratings from teachers, two from parents and four from both. Different types of questionnaires were utilised in different studies, with the Strengths and Difficulties Questionnaire (SDQ) (hyperactivity/inattention scale) being the most common (n = 3), followed by the Swanson, Nolan, and Pelham (SNAP- IV) Questionnaire (n = 2). Other measures included the Autism, Tics, ADHD and other Comorbidities Inventory (A-TAC), Conner’s Teacher Rating Scale (CTRS), Perceived Competence Scale (PCS), Children’s Behavioural Scale (CBS) and two unspecified scales. The risk of bias was assessed using the NOS with three scoring as high methodological quality and six as moderate. A RAE was observed for teacher ratings of ADHD symptoms in two of the three studies investigating teacher ratings only, with positive findings showing up to a 3.6-fold increase in the youngest quartile [14, 21]. Three studies showed a RAE on teachers’ ratings, with up to threefold increase in reported ADHD symptoms by teachers, but not parents’ [13, 22, 23], and one study reported a association with both, by estimating a 3.5% and 1.1% difference in teacher and parental ratings respectively [23]. There was no association between relative age and parent ratings for ADHD using the A-TAC inventory [24], while one study using the hyperactivity/inattention section of SDQ reported an association between younger relative age and parental ratings [25]. One study found an association between younger relative age and higher scores for ADHD symptoms for both teacher and parent ratings (75% and 54% higher respectively)[26].

### ADHD clinical diagnosis

Seventeen papers used clinical diagnosis of ADHD as an outcome measure, with twelve using database records and five parent-reported clinician diagnoses [13, 23, 26–28]. Ten studies were of high methodological quality and seven of moderate. Fifteen studies found a relative age effect on the likelihood of ADHD diagnosis [1, 2, 10, 13, 23, 24, 27–35], and two found no association [26, 36].

### ADHD medications

Seventeen papers used prescription of ADHD medications, almost all derived from administrative databases, apart from two papers relying on parent-reported ADHD medications [13, 23]. Ten studies were of high methodological quality on NOS, six moderate and one low quality. Fourteen of the studies demonstrated a relative age effect for ADHD medication prescriptions, [2, 23, 27, 30–32, 34, 37–42] with three studies finding no association [13, 43, 44]. Two studies investigated prescribed medication in countries with high rates of held-back children, accounting for children with delayed school entry, by collecting data from educational databases [11, 42]. Both studies showed that children who were the eldest within the school year, because they had been held-back, were more likely to be prescribed ADHD medications than their younger classmates who had entered school when first eligible [11, 42].

### Meta-analysis

**Fig. 2:**
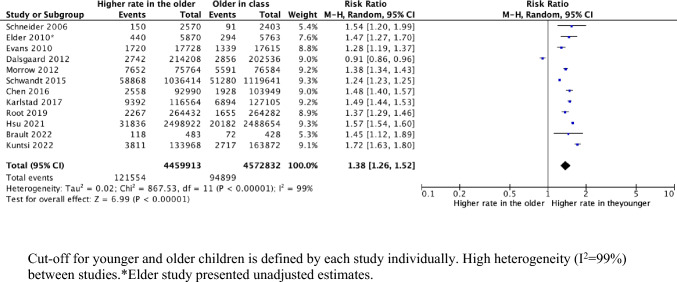
Number of children receiving a diagnosis of ADHD (events), comparing the and younger and older children within the school year

**Fig. 3:**
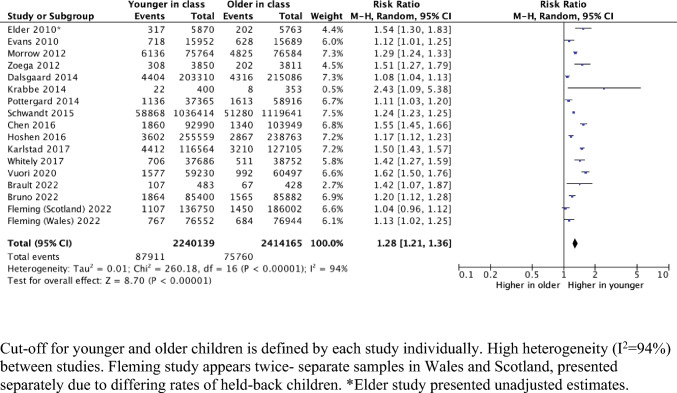
Number of children receiving ADHD medications (events), comparing the younger and older children within the school year

For ADHD diagnosis and medication, twenty-three studies were included in the meta-analysis. The rest could not be added due to: case control study design (n = 1) [24] or presenting insufficient data (n = 3) [26, 33, 34]. Corresponding authors were contacted to provide supplementary data to allow for inclusion in the meta-analysis (n = 6), and sufficient data were available and provided by one. For two papers, although authors did not provide supplementary data, this was retrieved from a previous meta-analysis in 2018 [3]. Two separate meta-analyses were conducted for studies investigating clinical diagnosis and medication prescription in relation to children’s relative age within school year. However, both showed high levels of heterogeneity (I^2^ = 99%, I^2^ = 94% respectively) (Figs. 2, 3).

Younger relative age was associated with ADHD diagnosis and medication, with RR of 1.38 (95% CI 1.36, 1.52) and 1.28 (95% CI 1.21, 1.36) respectively. This means that children who are younger relative to their peers within the same school year are 38% more likely to receive an ADHD diagnosis and 28% more likely to be prescribed ADHD medications. Risk ratio plots (Figs. 2, 3) showed the risk ratio estimates from individual studies. All included studies investigating ADHD diagnosis showed higher risk ratios for younger children in their school year compared with their older classmates, apart from one in Denmark, which found an opposite effect with RR 0.91 (95%CI 0.86,0.96) [36]. (Fig. 2) For prescribed medication, all studies showed higher risk of ADHD medication prescriptions for younger relative age, except for one study in Scotland [11]. (Fig. 3).

For studies investigating ADHD symptoms, a meta-analysis was not possible due to the variability in rating scales used, differences in the informant, and methodological differences (Table 2).

**Table 2:** Summary of characteristics and main results of included studies investigating ASD

Study	Years studied	Sample size (n)	Country	Data source	Reported Social, Demographic Characteristics^a^	Ages studied	School starting age/ Cut-off date	ADHD Symptoms/ Diagnosis/Medication	Symptoms measure	Clinical diagnosis definition	Medication definition	Time Comparison	RAE (Relative Age Effect)	Strength of RAE	NOS^b^
Hsu (2021)	1995–2013	18 902	Taiwan	National Health Insurance Research Database (NHIRD)	Not reported	Not specified	Cut-off date August 31st	Diagnosis	n/a	DSM-5 diagnosis of ASD by a board-certified psychiatrist, as recorded on NHIRD	n/a	Monthly	Yes	Children born in August had RR of 1.1 for an ASD diagnosis, p < 0.001. (Adjusted^d^)	7/8
Chen (2021)	2001–2011	24,386	Taiwan	National Health Research Institute Database (NHRID)	Not reported^c^	3–17	Cut-off date August 31st	Diagnosis	n/a	ICD diagnosis of ASD by board-certified psychiatrists, as recorded on NHIRD	n/a	Monthly	Yes	Children born in August had OR 1.23 [95% CI 1.16–1.31] for ASD diagnosis, p < 0.001. (Adjusted^d^)	7/8

aThere was no consistency in the reported sociodemographic characteristics of the total sample of included studies. bNOS Newcastle Ottawa Score. cAuthors adjusted their analysis for some sociodemographic characteristics. dAuthors adjusted for variables of their choice. List of individual variables adjusted for in each study can be found in individual papers

### Autism spectrum disorder

The two studies investigating the effect of relative age on diagnosis of ASD were both from Taiwan [5, 34]. In both studies, children who were the youngest in their school year were more likely to be diagnosed with ASD than those who were the eldest. Both studies were found to be of high methodological quality, scoring seven on the NOS. One considered the relative age effect on multiple neurodevelopmental disorders and reported a highly pronounced drop in diagnosis rate between the birth months of August and September, i.e. the month immediately before and after the school entry cut-off, for both ADHD and ASD with the relationship being most pronounced for ADHD [5].

## Discussion

This systematic review summarises the available evidence on the influence of relative age on the rating of ADHD symptoms by teachers or parents, revealing a discrepancy between the impact of relative age on teacher and parent ratings. These findings contribute to our understanding of this topic by showing that teacher ratings of ADHD symptoms were influenced by relative age; in contrast, parent ratings showed no or weak association with relative age [23, 24]. Our review also confirms findings from the previous literature [3, 4] by incorporating data from more recent studies, showing that the effect of relative age on clinical ADHD diagnosis and prescribed medication has persisted. Furthermore, we extend previous systematic reviews by investigating the relative age effect in ASD, a different neurodevelopmental disorder usually diagnosed in childhood [45].

The presence of ADHD symptoms in children as reported by teachers and parents, does not automatically translate to a formal diagnosis. Distinguishing between adult-reported symptoms and the diagnostic process allowed us to explore how relative age influences symptom interpretation, independent of diagnosis. Improved knowledge about how teachers and parents perceive and report ADHD symptoms is important as both are essential informants about a child’s ADHD-type behaviours in different settings [13, 46]. Overall, our results show that teacher ratings for ADHD-related symptoms are more influenced by relative age, in contrast to parent ratings. This difference between parent and teacher reporting of ADHD symptoms could be influenced by several factors. The higher demands and limited flexibility of the school environment, the presence of large numbers of peers to compare the child with, and the less close and shorter duration of the relationship of teacher to child compared with parent to child could all cause this observational bias in teacher ratings [3, 14, 26]. The limited classroom-specific support strategies for teachers to help relatively younger children with ADHD symptoms meet classroom expectations may also influence their assessments of ADHD symptoms. Parents may also be subject to social desirability bias towards their child, potentially overlooking certain behaviours. Teacher perceptions and susceptibility to relative age bias may impact a child’s referral and diagnostic process. Teachers are more likely to identify ADHD symptoms in younger children in the school year and give higher scores on symptom scales, which are then taken into account by clinicians when doing a diagnostic assessment. Conversely, teachers might also miss ADHD symptoms in relative older children in the class as they are being referenced against their younger and slightly less mature classmates [23].

In terms of diagnosis and prescriptions, our results overall confirm an association between younger relative age and a clinical diagnosis of ADHD or prescribed medication. The strength of this association showed high heterogeneity for both outcomes. This could be explained by methodological factors such as different ways of measuring exposure and outcomes, variability in sample characteristics, sample sizes and cut-offs for ‘oldest’ and ‘youngest’ in the year; educational differences such as different curriculums, systems and policies including but not limited to rates of delayed school entry, rates of repeating school year due to failure to progress, absolute age at starting school, school classroom size; and cultural differences such as societal attitudes towards neurodevelopmental disorders or expectations around conformity and educational achievements. International variations in diagnostic and prescription practices, including access to services and who can make a clinical diagnosis, likely contribute to the observed heterogeneity, reflecting discrepancies in ADHD identification and medication use rates across countries. Such cross-cultural differences in ADHD diagnostic and treatment guidelines should be considered when interpreting the findings of international studies [3, 27, 44]. Most studies did not consistently report sociodemographic characteristics of their total sample; although six studies adjusted their analysis for some sociodemographic characteristics as potential confounders. Due to many studies relying on nationwide prescription or health record databases, data collected by primary authors were often representative of the clinically relevant populations of the area.

A persistent relative age effect was found for studies published since 2018 (when the previous systematic review was conducted) for both diagnosis and medication [1, 5, 13, 29]. This phenomenon has been documented in the literature over the past decade, so one might expect that clinicians to factor relative age into assessments, however, there is little evidence that this has occurred [37]. One reason could be the lack of guidance on how to best account for relative age in the diagnostic process, as international guidelines such as NICE and American Academy of Pediatrics do not incorporate relative age considerations [46, 47]. Other factors could be diagnostic uncertainty, clinicians’ time pressure, reliance on subjective evaluations or over-reliance on standardised questionnaires, limiting the ability perform age-matched comparisons for younger children.

The relative age effect on ADHD shows a pronounced impact in younger children attending primary school, with peak age varying in individual studies, but overall gradually diminishing through adolescence. This observation suggests that actual age and developmental expectations significantly influence ADHD identification, with early schooling years witnessing the greatest disparities [1, 2, 32, 33]. Existing literature has discounted the presence of a seasonal effect on ADHD, as variations do not align with specific seasons but shift according to local school entrance policies [38]. Previous meta-analyses have speculated that more flexible school entry could reduce the relative age effect [3, 4]. However, two recent studies found that children who were held back a year, entering school relatively later than their classmates, were more likely to be prescribed ADHD medication [11, 42]. The authors explained this may be due to systematic differences in children with delayed school entry, such as having more complex special developmental needs [42] or parents who worry more about perceived relative immaturity and neurodevelopmental diagnoses [11]. However, these findings suggest that changes to school entry policy may not have the desired effect of reducing the relative age effect. Importantly, families of higher socioeconomic status are more likely to afford deferring their child’s school entry while less affluent families are more likely to have children in the youngest school year cohort [11]. This introduces a social inequity aspect to the disparity in diagnostic rates in areas with flexible school entry policies.

While the relative age effect is well documented in ADHD, data for other neurodevelopmental disorders are still emerging. Only two studies, both from the same country using the same data source, were identified investigating the relative age effect in ASD diagnosis and children who were the youngest in their school year were more likely to be diagnosed with ASD compared with their older classmates. The reasons behind this are not clear but it is possible that immature speech or social skills of relatively younger children may be interpreted as traits of autism by referrers [5, 48]. Although the timing of identification of characteristics for many autistic children takes place before they reach school age [49, 50], no information on the age of autism diagnosis was available in the two studies to comment on the differences between autistic children diagnosed before school age and those after.

### Strengths and limitations

A strength of this review was the systematic search of the available literature across seven databases by two independent reviewers and quality of studies was assessed as high or moderate for all except one study. Studies from eighteen countries were included, collecting data from diverse settings. However, there is a risk of potential overlap in populations across studies from the same countries and using similar databases, especially among the Taiwanese studies, which should be taken into account when interpreting the weight of our findings. For studies investigating ADHD-related symptoms, there was wide variability in chosen assessment tools, leading to challenges in comparing the results between studies. Even though our meta-analysis aimed at combining quantitative data from studies that offered sufficient detail, the high heterogeneity across studies, (despite our attempts to explain the reasons behind it), made it challenging to generalize our findings reliably. Additionally, not all retrieved studies could be included in the meta-analysis due to the way data were analysed and presented, meaning some studies with large datasets were excluded from the quantitative synthesis. Although searches were comprehensive there is a small risk of publication bias, as smaller studies or ones with negative results may have not been published and so might have been missed from our database search, potentially causing an over-estimation of the studied effect. Pervasive developmental disorder not otherwise specified (PDD-NOS) was not included in our search criteria, which could be seen as a limitation. For studies investigating teacher ratings, there was no information on the training and experience of individual teachers in identifying ADHD symptoms or on how long they had known the child.

### Clinical and research implications

Teacher ratings form an important part of ADHD assessments, and so it is important to understand the effect of relative age on their perception of what are normative or immature behaviours. Clinicians would benefit from collaborative involvement of parents and teachers in their assessments, whilst taking into account the possible differences in the relative age effect bias of these two informants. Despite the relative age effect being previously described in the literature, this has not translated into a change in clinical practice for diagnosis or medication prescribing, although a conclusion around the magnitude of the relative age effect is difficult to draw given the level of heterogeneity observed. It will be useful to incorporate this phenomenon in the clinical guidelines and training of healthcare professionals specialising in neurodevelopmental disorders as well as teachers to help them think critically about children’s symptoms during their assessments. Importantly, diagnosis of ADHD in relatively younger children is no more likely to decrease in persistence than diagnoses in relatively older children and so the relative age effect should not necessarily deter clinicians from diagnosing relatively younger children with ADHD [51]. Referrers and clinicians should consider the possibility that the relative age effect may be leading to a decreased likelihood of older children in the class being identified with ADHD symptoms [51]. Systematically considering contributing factors like relative immaturity as part of a child’s presentation is important to improve accuracy of ADHD diagnosis and subsequent appropriate treatment with medications.

Given the prevalence of ADHD [52], addressing the diagnostic challenges and accounting for biases becomes increasingly relevant. From a research perspective, a more standardised methodology (e.g. choice of measures) across future studies would allow for a more reliable quantitative analysis due to more comparable results, which was not possible in this work.

In terms of educational implications, studies investigating the effect of delaying school entry on children’s likelihood of being prescribed ADHD medication have found that held-back children were more likely to be treated for ADHD. As this work is crucial to informing educational policies, further research on the impact of flexible school entry would be valuable, as current evidence is limited.

Further research is necessary to replicate and extend the current limited findings on ASD, investigating if this effect is also present in other countries and healthcare settings.

## Conclusion

The relative age effect in ADHD, despite being well documented within research for over a decade, is still present in diagnostic and prescribing practice across the world. This review extends previous findings by showing consistent evidence across studies that compared to parent ratings, teacher ratings of ADHD-related symptoms are more influenced by relative age. Emerging findings also suggest it may be a factor in the diagnosis of other neurodevelopmental disorders such as ASD.

## Supplementary Information

Below is the link to the electronic supplementary material.

Supplementary file1 (DOCX 26 KB)

Supplementary file2 (DOCX 22 KB)

Supplementary file3 (DOCX 21 KB)

Supplementary file4 (DOCX 17 KB)
