# Supplementary material for: Systematic review and meta-analysis: relative age in attention-deficit/ hyperactivity disorder and autism spectrum disorder
Source: Eur Child Adolesc Psychiatry. 2024 May 20;34(2):381–401. doi: 10.1007/s00787-024-02459-x (PMC11868292; doi:10.1007/s00787-024-02459-x)
Supplement: Supplementary file 2 — Supplementary file2 (DOCX 22 KB) [file 787_2024_2459_MOESM2_ESM.docx]

Systematic Review and Meta-Analysis: Relative age in Attention-Deficit/ Hyperactivity Disorder and Autism Spectrum Disorder

ECAP

Eleni Frisira^1^, Josephine Holland^1^, Kapil Sayal^1^

^1^Institute of Mental Health, School of Medicine, Mental Health and Clinical Neurosciences, University of Nottingham, Nottingham, UK

[mszjkh@exmail.nottingham.ac.uk](mailto:mszjkh@exmail.nottingham.ac.uk)

Online Resource 2: Evidence search: Relative age and ADHD. Naomi Thorpe. 23 August 2022. Nottinghamshire Healthcare NHS Foundation Trust, Library and Knowledge Services.

**Database Searching**

| **Name of Database** | **Platform** | **Date searched** | **# of results** |
| --- | --- | --- | --- |
| Medline | Ovid MEDLINE® ALL <1946 to August 22, 2022> | 23^rd^ August 2022 | 240 |
| Embase | Ovid Embase <1974 to 2022 August 22> | 23^rd^ August 2022 | 315 |
| PsycInfo | Ovid PsycINFO <1806 to August Week 3 2022> | 23^rd^ August 2022 | 252 |
| Web of Science Core Collection | Clarivate | 23^rd^ August 2022 | 355 |
| ERIC | EBSCOhost Research Databases | 23^rd^ August 2022 | 27 |
| Psychology and Behavioral Sciences Collection | EBSCOhost Research Databases | 23^rd^ August 2022 | 74 |
| Cochrane Library | <http://www.cochranelibrary.com/> | 23^rd^ August 2022 | 61 |
|  | | | |
| **Total number of results** | | | 1324 |
| **Duplicates found** | | | 684 |
| **Results after duplicates removed** | | | 640 |

**Search history for databases for published literature**

**Platform and database**: Ovid MEDLINE® ALL <1946 to August 22, 2022>

**Date searched**: 23-08-2022

--------------------------------------------------------------------------------

| **#** | **Search term(s)** | **Result(s)** |
| --- | --- | --- |
| 1 | (relative adj (age* or matur* or immatur*)).tw,kw,kf. | 1142 |
| 2 | ("young for grade*" or "young for year*").tw,kw,kf. | 1789 |
| 3 | ("old for grade*" or "old for year*").tw,kw,kf. | 1695 |
| 4 | ("influence of age" or "birthdate effect*" or "birth date effect*" or "age effect*" or "season of birth*" or "age position*").tw,kw,kf. | 15536 |
| 5 | or/1-4 | 19593 |
| 6 | Attention Deficit Disorder with Hyperactivity/ or Hyperkinesis/ | 37300 |
| 7 | (adhd or addh or adhs or impulsiv* or inattentiv* or inattention* or hyperactiv* or hyperkinesis*).tw,kw,kf. | 97531 |
| 8 | ((attention* or behav*) adj3 (defic* or dysfunc* or disorder*)).tw,kw,kf. | 74893 |
| 9 | ((disrupt* or defian*) adj3 (disorder* or behav*)).tw,kw,kf. | 10684 |
| 10 | or/6-9 | 151070 |
| 11 | 5 and 10 | 248 |
| 12 | limit 11 to english language | 240 |

***************************

**Platform and database**: Ovid Embase <1974 to 2022 August 22>

**Date searched**: 23-08-2022

--------------------------------------------------------------------------------

| **#** | **Search term(s)** | **Result(s)** |
| --- | --- | --- |
| 1 | (relative adj (age* or matur* or immatur*)).tw,kw,kf. | 1181 |
| 2 | ("young for grade*" or "young for year*").tw,kw,kf. | 2837 |
| 3 | ("old for grade*" or "old for year*").tw,kw,kf. | 2885 |
| 4 | ("influence of age" or "birthdate effect*" or "birth date effect*" or "age effect*" or "season of birth*" or "age position*").tw,kw,kf. | 18925 |
| 5 | or/1-4 | 25177 |
| 6 | Attention Deficit Hyperactivity Disorder/ or Hyperkinesia/ | 8702 |
| 7 | (adhd or addh or adhs or impulsiv* or inattentiv* or inattention* or hyperactiv* or hyperkinesis*).tw,kw,kf. | 133347 |
| 8 | ((attention* or behav*) adj3 (defic* or dysfunc* or disorder*)).tw,kw,kf. | 100593 |
| 9 | ((disrupt* or defian*) adj3 (disorder* or behav*)).tw,kw,kf. | 13555 |
| 10 | or/6-9 | 200921 |
| 11 | 5 and 10 | 328 |
| 12 | limit 11 to english language | 315 |

***************************

**Platform and database**: Ovid APA PsycInfo <1806 to August Week 3 2022>

**Date searched**: 23-08-2022

--------------------------------------------------------------------------------

| **#** | **Search term(s)** | **Result(s)** |
| --- | --- | --- |
| 1 | (relative adj (age* or matur* or immatur*)).tw,kw,kf. | 428 |
| 2 | ("young for grade*" or "young for year*").tw,kw,kf. | 384 |
| 3 | ("old for grade*" or "old for year*").tw,kw,kf. | 601 |
| 4 | ("influence of age" or "birthdate effect*" or "birth date effect*" or "age effect*" or "season of birth*" or "age position*").tw,kw,kf. | 6131 |
| 5 | or/1-4 | 7346 |
| 6 | exp Attention Deficit Disorder/ or Hyperkinesis/ | 34068 |
| 7 | (adhd or addh or adhs or impulsiv* or inattentiv* or inattention* or hyperactiv* or hyperkinesis*).tw,kw,kf. | 80715 |
| 8 | ((attention* or behav*) adj3 (defic* or dysfunc* or disorder*)).tw,kw,kf. | 80713 |
| 9 | ((disrupt* or defian*) adj3 (disorder* or behav*)).tw,kw,kf. | 14280 |
| 10 | or/6-9 | 130020 |
| 11 | 5 and 10 | 278 |
| 12 | limit 11 to english language | 252 |

***************************

**Platform and Database:** Clarivate Web of Science Core Collection

**Date searched:** 23-08-2022

--------------------------------------------------------------------------------

| **#** | **Search term(s)** | **Result(s)** |
| --- | --- | --- |
| #1 | relative NEAR/1 (age* OR matur* OR immatur*) (TOPIC) | 8,927 |
| #2 | "young for grade*" OR "young for year*" (TOPIC) | 10 |
| #3 | "old for grade*" OR "old for year*" (TOPIC) | 20 |
| #4 | "influence of age" OR "birthdate effect*" OR "birth date effect*" OR "age effect*" OR "season of birth*" OR "age position*" (TOPIC) | 18,238 |
| #5 | #1 OR #2 OR #3 OR #4 | 26,546 |
| #6 | adhd OR addh OR adhs OR impulsiv* OR inattentiv* OR inattention* OR hyperactiv* OR hyperkinesis* (TOPIC) | 160,834 |
| #7 | (attention* OR behav*) NEAR/3 (defic* OR dysfunc* OR disorder*) (TOPIC) | 106,987 |
| #8 | (disrupt* or defian*) NEAR/3 (disorder* or behav*) (TOPIC) | 16,906 |
| #9 | #6 OR #7 OR #8 | 229562 |
| #10 | #5 AND #9 | 372 |
| #11 | #5 AND #9  Refined By: Languages: English | 355 |

***************************

**Platform and database:** EBSCOhost Research Databases ERIC

**Date searched:** 23-08-2022

--------------------------------------------------------------------------------

| **#** | **Search term(s)** | **Result(s)** |
| --- | --- | --- |
| S1 | TI ( relative N1 (age* OR matur* OR immatur*) ) OR AB ( relative N1 (age* OR matur* OR immatur*) ) | 330 |
| S2 | TI ( "young for grade*" OR "young for year*" ) OR AB ( "young for grade*" OR "young for year*" ) | 134 |
| S3 | TI ( "old for grade*" OR "old for year*" ) OR AB ( "old for grade*" OR "old for year*" ) | 104 |
| S4 | TI ( "influence of age" OR "birthdate effect*" OR "birth date effect*" OR "age effect*" OR "season of birth*" OR "age position*" ) OR AB ( "influence of age" OR "birthdate effect*" OR "birth date effect*" OR "age effect*" OR "season of birth*" OR "age position*" ) | 603 |
| S5 | S1 OR S2 OR S3 OR S4 | 1120 |
| S6 | (DE "Attention Deficit Hyperactivity Disorder") OR (DE "Hyperactivity") | 6129 |
| S7 | TI ( adhd OR addh OR adhs OR impulsiv* OR inattentiv* OR inattention* OR hyperactiv* OR hyperkinesis* ) OR AB ( adhd OR addh OR adhs OR impulsiv* OR inattentiv* OR inattention* OR hyperactiv* OR hyperkinesis* ) | 8007 |
| S8 | TI ( (attention* OR behav*) N3 (defic* OR dysfunc* OR disorder*) ) OR AB ( (attention* OR behav*) N3 (defic* OR dysfunc* OR disorder*) ) | 10008 |
| S9 | TI ( (disrupt* OR defian*) N3 (disorder* OR behav*) ) OR AB ( (disrupt* OR defian*) N3 (disorder* OR behav*) ) | 2907 |
| S10 | S6 OR S7 OR S8 OR S9 | 16840 |
| S11 | S5 AND S10 | 27 |

***************************

**Platform and database:** EBSCOhost Research Databases Psychology and Behavioral Sciences Collection

**Date searched:** 23-08-2022

--------------------------------------------------------------------------------

| **#** | **Search term(s)** | **Result(s)** |
| --- | --- | --- |
| S1 | TI ( relative N1 (age* OR matur* OR immatur*) ) OR AB ( relative N1 (age* OR matur* OR immatur*) ) | 299 |
| S2 | TI ( "young for grade*" OR "young for year*" ) OR AB ( "young for grade*" OR "young for year*" ) | 157 |
| S3 | TI ( "old for grade*" OR "old for year*" ) OR AB ( "old for grade*" OR "old for year*" ) | 95 |
| S4 | TI ( "influence of age" OR "birthdate effect*" OR "birth date effect*" OR "age effect*" OR "season of birth*" OR "age position*" ) OR AB ( "influence of age" OR "birthdate effect*" OR "birth date effect*" OR "age effect*" OR "season of birth*" OR "age position*" ) | 1059 |
| S5 | S1 OR S2 OR S3 OR S4 | 1552 |
| S6 | (DE "ATTENTION-deficit hyperactivity disorder") OR (DE "HYPERKINESIA") | 6024 |
| S7 | TI ( adhd OR addh OR adhs OR impulsiv* OR inattentiv* OR inattention* OR hyperactiv* OR hyperkinesis* ) OR AB ( adhd OR addh OR adhs OR impulsiv* OR inattentiv* OR inattention* OR hyperactiv* OR hyperkinesis* ) | 13668 |
| S8 | TI ( (attention* OR behav*) N3 (defic* OR dysfunc* OR disorder*) ) OR AB ( (attention* OR behav*) N3 (defic* OR dysfunc* OR disorder*) ) | 13720 |
| S9 | TI ( (disrupt* OR defian*) N3 (disorder* OR behav*) ) OR AB ( (disrupt* OR defian*) N3 (disorder* OR behav*) ) | 2580 |
|  | S6 OR S7 OR S8 OR S9 | 22777 |
|  | S5 AND S10 | 74 |

***************************

**Platform and database:** Cochrane Library Cochrane Database of Systematic Reviews

**Date searched:** 23-08-2022

--------------------------------------------------------------------------------

| **#** | **Search term(s)** | **Result(s)** |
| --- | --- | --- |
| #1 | (relative NEAR/1 (age OR mature OR immature)):ti,ab,kw (Word variations have been searched) | 7349 |
| #2 | ("young for grade" OR "young for year"):ti,ab,kw (Word variations have been searched) | 0 |
| #3 | ("old for grade" OR "old for year"):ti,ab,kw (Word variations have been searched) | 0 |
| #4 | ("influence of age" OR "birthdate effect" OR "birth date effect" OR "age effect" OR "season of birth" OR "age position"):ti,ab,kw (Word variations have been searched) | 940 |
| #5 | #1 OR #2 OR #3 OR #4 | 8207 |
| #6 | MeSH descriptor: [Autistic Disorder] explode all trees | 3055 |
| #7 | MeSH descriptor: [Autism Spectrum Disorder] explode all trees | 626 |
| #8 | (adhd OR addh OR adhs OR impulsive OR inattentive OR inattention OR hyperactive OR hyperkinesis):ti,ab,kw (Word variations have been searched) | 12851 |
| #9 | ((attention OR behaviour) NEAR/3 (deficiency OR dysfunction OR disorder)):ti,ab,kw (Word variations have been searched) | 13068 |
| #10 | ((disruptive OR defiant) NEAR/3 (disorder OR behaviour)):ti,ab,kw (Word variations have been searched) | 1461 |
| #11 | #6 OR #7 OR #8 OR #9 OR #10 | 20181 |
| #112 | #5 AND #11 | 61 |

***************************
