# Supplementary material for: Systematic review and meta-analysis: relative age in attention-deficit/ hyperactivity disorder and autism spectrum disorder
Source: Eur Child Adolesc Psychiatry. 2024 May 20;34(2):381–401. doi: 10.1007/s00787-024-02459-x (PMC11868292; doi:10.1007/s00787-024-02459-x)
Supplement: Supplementary file 4 — Supplementary file4 (DOCX 17 KB) [file 787_2024_2459_MOESM4_ESM.docx]

Systematic Review and Meta-Analysis: Relative age in Attention-Deficit/ Hyperactivity Disorder and Autism Spectrum Disorder

ECAP

Eleni Frisira^1^, Josephine Holland^1^, Kapil Sayal^1^

^1^Institute of Mental Health, School of Medicine, Mental Health and Clinical Neurosciences, University of Nottingham, Nottingham, UK

[mszjkh@exmail.nottingham.ac.uk](mailto:mszjkh@exmail.nottingham.ac.uk)

Online Resource 4: Individual study breakdown for assessment of risk of bias using Newcastle-Ottawa Scale.

| **Study** | **Selection (S) Comparability (C) Outcome (O)** | **Total score** | **Quality Category** |
| --- | --- | --- | --- |
| Dalsgaard (2012) | S3/4, C2/2, O1/1 | 6/7 | Moderate |
| Sayal (2017) | S3/4, C2/2, O2/2 | 7/8 | High |
| Bonati (2018) | S2/4, C2/2, O2/2 | 6/8 | Moderate |
| Root (2019) | S3/4, C2/2, O2/2 | 7/8 | High |
| Hsu (2021) | S3/4, C2/2, O2/2 | 7/8 | High |
| Kuntsi (2022) | S3/4, C0/2, O2/2 | 5/8 | Moderate |
| Schneider (2006) | S3/4, C2/2, O0/2 | 5/8 | Moderate |
| Evans (2010) | S2/4 C2/2, O1/2 | 5/8 | Moderate |
| Morrow (2012) | S3/4, C2/2, O2/2 | 7/8 | High |
| Schwandt (2015) | S4/4, C2/2, O2/2 | 8/8 | High |
| Chen (2016) | S3/4, C2/2, O2/2 | 7/8 | High |
| Karlstad (2017) | S3/4, C2/2, O2/2 | 7/8 | High |
| Chen (2021) | S3/4, C2/2, O2/2 | 7/8 | High |
| Diefenbach (2021) | S4/4, C2/2, O2/3 | 8/9 | High |
| Halldner (2014) | S3/4, C2/2, O3/3 | 7/8 | High |
| Elder (2010) | S2/4, C2/2, O1/2 | 5/8 | Moderate |
| Brault (2022) | S2/4, C2/2, O2/3 | 6/9 | Moderate |
| Dalsgaard (2013) | S3/4, C2/2, O2/2 | 7/8 | High |
| Hoshen (2016) | S3/4, C2/2, O 3/3 | 8/9 | High |
| Zoega (2012) | S3/4, C1/2, O2/2 | 6/8 | Moderate |
| Pottergard (2014) | S3/4, C2/2, O2/2 | 7/8 | High |
| Whitely (2017) | S3/4, C1/2, O 2/2 | 6/8 | Moderate |
| Vuori (2020) | S3/4, C1/2, O2/2 | 6/8 | Moderate |
| Bruno (2022) | S3/4, C2/2, O2/2 | 7/8 | High |
| Fleming (2022) | S3/4, C2/2, O2/2 | 7/8 | High |
| Gökçe (2016) | S2/4, C2/2, O1/3 | 5/9 | Moderate |
| Wienen (2018) | S2/4, C 2/2, O 2/3 | 6/9 | Moderate |
| Oner (2019) | S1/3, C2/2, O 1/1 | 4/6 | Low |
| Broughton (2022) | S4/4, C2/2, O2/3 | 8/9 | High |
| Furzer (2022) | S3/4, C1/2, O1/3 | 5/9 | Moderate |
